# The European DISABKIDS project: development of seven condition-specific modules to measure health related quality of life in children and adolescents

**DOI:** 10.1186/1477-7525-3-70

**Published:** 2005-11-13

**Authors:** Rolanda M Baars, Clare I Atherton, Hendrik M Koopman, Monika Bullinger, Mick Power, the DISABKIDS group*

**Affiliations:** 1 Department of Paediatrics, Leiden University Medical Center, Leiden, The Netherlands; 2 Department of Psychiatry, University of Edinburgh, Edinburgh, UK; 3 Department of Medical Psychology, University of Hamburg, Hamburg, Germany; 4 Section of Clinical and Health Psychology, University of Edinburgh, Royal Edinburgh Hospital, Morningside Park, Edinburgh EH 10 5HF, United Kingdom

## Abstract

**Background:**

The European DISABKIDS project aims to enhance the Health Related Quality of Life (HRQoL) of children and adolescents with chronic medical conditions and their families. We describe the development of the seven cross-nationally tested condition-specific modules of the European DISABKIDS HRQoL instrument in a population of children and adolescents. The condition-specific modules are intended for use in conjunction with the DISABKIDS chronic generic module.

**Methods:**

Focus groups were used to construct the pilot version of the DISABKIDS condition-specific HRQoL modules for asthma, juvenile idiopathic arthritis, atopic dermatitis, cerebral palsy, cystic fibrosis, diabetes and epilepsy. Analyses were conducted on pilot test data in order to construct field test versions of the modules. A series of factor analyses were run, first, to determine potential structures for each condition-specific module, and, secondly, to select a reduced number of items from the pilot test to be included in the field test. Post-field test analyses were conducted to retest the domain structure for the final DISABKIDS condition-specific modules.

**Results:**

The DISABKIDS condition-specific modules were tested in a pilot study of 360 respondents, and subsequently in a field test of 1152 respondents in 7 European countries. The final condition-specific modules consist of an 'Impact' domain and an additional domain (e.g. worry, stigma, treatment) with between 10 to 12 items in total. The Cronbach's alpha of the final domains was found to vary from 0.71 to 0.90.

**Conclusion:**

The condition-specific modules of the DISABKIDS instrument were developed through a step-by-step process including cognitive interview, clinical expertise, factor analysis, correlations and internal consistency. A cross-national pilot and field test were necessary to collect these data. In general, the internal consistency of the domains was satisfactory to high. In future, the DISABKIDS instrument may serve as a useful tool with which to assess HRQoL in children and adolescents with a chronic condition. The condition-specific modules can be used in conjunction with the DISABKIDS chronic generic module.

## Background

The last few decades have seen an increase in the amount of constructed Health Related Quality of Life (HRQoL) questionnaires for use with children and adolescents [1,2]. Although a number of questionnaires have been used for evaluative studies the questionnaires are only occasionally used in paediatric clinical trials or clinical practice [3-5]. The expectation is that the implementation of HRQoL questionnaires will increase once a number of aspects of HRQoL research are improved.

One area of improvement concerns the need for valid cross-national questionnaires for use in international research [6-8]. Most questionnaires have been developed in one country and are then translated for use in other countries (sequential approach) [9]. This is thought to have its limitations as true compatibility is not necessarily reached [8,10]. A preferred design for the development of cross-national questionnaires is to construct a questionnaire in several countries through a simultaneous approach [8,9]. A questionnaire that was developed in simultaneous collaboration with different countries is the World Health Organization Quality of Life (WHOQOL) questionnaire, but it is only for use in adults [11].

Investigators have also suggested further improvement of HRQoL questionnaires by combining generic and condition-specific modules to offer sufficient detail in the assessment of HRQoL [12]. Generic questionnaires are generally used in HRQoL research and enable comparisons between groups of interest (i.e. different chronic medical conditions). Supplementing a generic module with a condition-specific module is suggested to provide additional information concerning a specific condition and has the potential to identify smaller changes important to research or clinical practice [12-14]. Examples of these are the 'How are you?' (HAY)-asthma [15,16] and the Paediatric Quality of Life Inventory (PedsQL™) [17,18], which both consist of a generic core scale with an additional asthma module.

However, thus far there were no HRQoL questionnaires that were developed in several countries simultaneously and consisted of a chronic generic and condition-specific module for use in children and adolescents with a variety of chronic medical conditions. The European DISABKIDS project aimed to provide in this need. The project was conducted simultaneously in collaboration with seven European countries and developed a series of modules to assess the HRQoL of children and adolescents who suffer from chronic medical conditions [19]. The unique combination consisted of the simultaneous cross-national development, the patient-derived bottom-up procedure, a two modular design and the inclusion of seven chronic conditions. This paper will illustrate the psychometric procedures that have been employed in the development of the condition-specific modules for the European DISABKIDS instrument. Results will be presented and limitations will be discussed. A pilot study was performed to test the basic domain structure and reduce the number of items. A larger field study was conducted to carry out the statistical analyses for the final version of the seven condition-specific DISABKIDS modules. The asthma-specific module will be described in more detail to illustrate the developmental process.

## Methods

**Figure 1:**
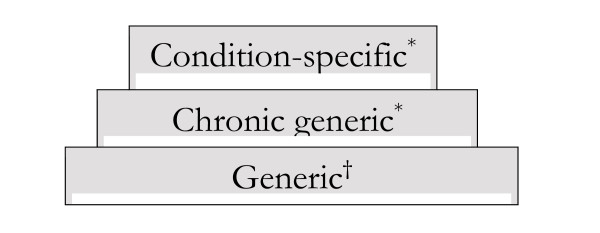
Modular design of the DISABKIDS* and KIDSCREEN^† ^instrument.

The DISABKIDS group has developed a European HRQoL instrument for children and adolescents with a chronic medical condition and their parents [19]. The project is a collaboration of seven European countries (Austria, France, Germany, Greece, the Netherlands, Sweden and the United Kingdom) and included seven chronic medical conditions: asthma, juvenile idiopathic arthritis (JIA), atopic dermatitis, cerebral palsy (CP), cystic fibrosis (CF), diabetes and epilepsy. The work was closely linked to the KIDSCREEN project, which is concerned with the development of a generic Quality of Life (QoL) questionnaire for children of the general population through a similar methodology [20,21]. The instruments devised by these two projects form a three level modular structure (Figure 1).

The generic module is provided by the KIDSCREEN project and is a QoL questionnaire, suitable for all children, regardless of whether they enjoy complete health or suffer from a chronic medical condition. This generic module creates the possibility of comparing children with a chronic condition to healthy children. The DISABKIDS project has provided the other two modules. One is referred to as the chronic generic module, which is suitable for use with children and adolescents who suffer from any chronic medical condition. It can compare HRQoL across different conditions while taking into account specific areas affected by a chronic condition [22]. The third level consists of a condition-specific module, one for every chronic condition studied in the DISABKIDS project. Each one concerns aspects related to a specific chronic condition and can only compare between data from patients with the same chronic condition. In practice children and adolescents with a chronic medical condition can complete all three modules as each provides different information.

**Figure 2:**
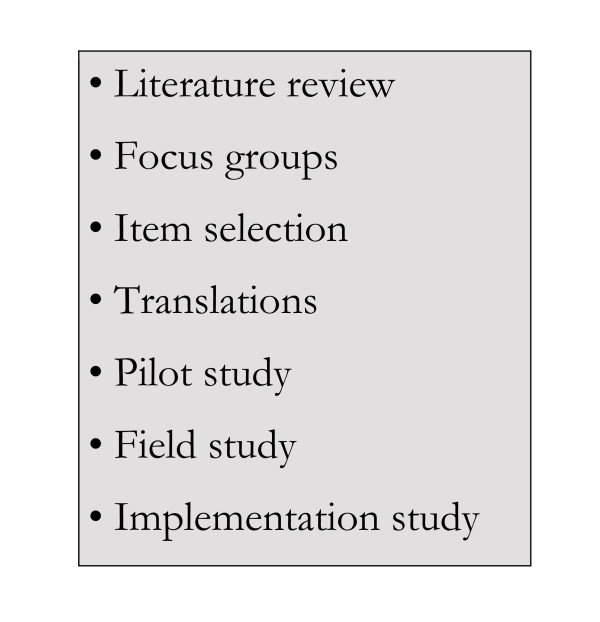
Work packages within the DISABKIDS project.

The DISABKIDS project has followed a stepwise methodology of questionnaire construction (Figure 2). Prior to the development of the instrument, an extensive literature review was conducted, and existing HRQoL questionnaires were reviewed in order to obtain an understanding of items in use. Central to the DISABKIDS project was the 'bottom-up' (patient-derived) nature of questionnaire construction, which was accomplished by involving children and adolescents with a chronic medical condition throughout the project. Focus groups and interviews were carried out in order to identify important HRQoL aspects from the perspective of children, adolescents and their parents. The participants were asked a series of semi-structured questions designed to facilitate discussion about their health and related quality of life issues. For example, "What kinds of things keep you healthy?" or "How does your condition affect you at school?". Participants were also asked to make suggestions as to what questions could be included in a QoL questionnaire suitable for others who suffer from the same condition as them. In this way the perspective of the child has been incorporated in order to ensure that the content of the questionnaire is directly relevant to the targeted age group [23].

HRQoL statements were selected from the collected qualitative data (focus group and interview transcripts) and merged into a data bank. Collected statements from each chronic condition group (asthma, epilepsy etc.) were then divided among the three modules of the instrument (Figure 1). Statements that were considered relevant to all children and adolescents, either healthy or suffering from a chronic condition were entered in the generic module and passed on to the KIDSCREEN project. General statements concerning chronic medical conditions were entered into the chronic generic module. Every disease specific statement was placed in the appropriate condition-specific module. To minimise the number of items, a redundancy scoring, item writing and card sorting procedure was constructed [22]. The card sorting procedure was performed by the DISABKIDS investigators and assisted in the final item selection and provided a preliminary domain structure for each module for use in the pilot study. The selected items were translated to the appropriate languages following general guidelines [24].

The aim of the pilot test was to select a reduced number of items to be included in the field test and to determine a preliminary scale structure within each condition-specific module. At this stage it was considered important to integrate both statistical and subjective data during the item selection process. This included the percentage of 'not applicable' and 'never' responses, a cognitive interview and the clinical judgment of clinicians and investigators. The cognitive interview provided detailed feedback on the relevance, age appropriateness and comprehensibility of the condition-specific items [25-27]. Children and adolescents were asked to rate the difficulty of each item and to rephrase each item in their own words. This feedback was used in conjunction with statistical analyses in order to make informed decisions about the item reduction [22]. The aim of the field test was to re-analyse the final domain structure of each condition-specific module and to calculate the internal consistency of each domain with data from a larger cross-national sample. Items were also examined for distribution of responses, frequency of non-response, ceiling and floor effects.

Children and adolescents between 8 and 16 years of age and their parents were asked to participate in the DISABKIDS pilot and field study, completing the instrument either at the hospital or at home. Data from the children and adolescents were used for the statistical analyses. Condition-specific modules were generally tested in at least two or more countries; only asthma was tested in all seven countries. Analysis of the condition-specific modules was carried out centrally (in the UK) to ensure that the item selection was done in a consistent way across all seven conditions. The analyses were performed separately for each condition-specific module and were carried out using SPSS Version 11.

## Results

### Pilot study

The pilot study instrument included the pilot version of the chronic generic module (100 items) and the pilot version of the condition-specific modules (between 26 and 44 items) (Table 1). The applied answer categories were never, seldom, quite often, very often and always, which were scored on a scale from 1 to 5 and an additional 'not applicable' option. The pilot study was conducted between May and August 2002.

**Table 1:** Number of items and participants (n = 360) in the pilot study for each condition-specific module

**Condition-specific modules**	**Number of items**	**Number of participants**	**Percentage of total sample (%)**
Asthma	32	132	37
Juvenile idiopathic arthritis	44	54	15
Atopic dermatitis	36	29	8
Cerebral palsy	26	21	6
Cystic fibrosis	38	28	8
Diabetes mellitus	28	59	16
Epilepsy	27	37	10

The sample for the pilot study consisted of 360 participating families. An equal number of boys and girls (48% and 52%) were included, mean age 12.5 (SD 2.55). The asthma group was the largest group of the sample (n = 132). Questionnaire data were only included when more than 60% of the items were completed, resulting in a total of 342 cases for the analyses. This left a few missing values, which were replaced with their series mean to evade losing additional data.

Various sources of data were systematically considered in the selection of items for domains. Some of the data were qualitative in nature, for example the clinical opinion gained from the relevant consultants participating in the project, cognitive interview feedback from the children and adolescents, and the investigator's judgement of the quality of the item. These qualitative aspects were used in conjunction with quantitative results from statistical analyses of the pilot test data (missing values, floor and ceiling effects). Some items were removed solely on the basis of qualitative data when 3 or more qualitative factors were identified as problematic (for example: not understood in the cognitive interview, too many missing values and not sufficiently related to HRQoL).

The structure of the condition-specific modules, as derived from the card sort procedure, was used as a starting point for the identification of domains within the pilot test modules. Item-domain correlations and reliability (Cronbach's alpha) were calculated for these scales. The domain structures resulting from the card sorting method were not generally robust in the statistical analyses. Therefore, principal components analysis with varimax rotation was conducted in order to identify possible new domains. The sample size was quite low for some conditions, and therefore factor analyses were viewed with caution.

An iterative procedure was followed in order to examine possible domain structures. Item groupings, found in the principal components analysis as being similar to those of the original domain structures (from the card sort procedure), were identified. On the basis of a similarity between these two methods, 3–6 items were selected per domain. A scale was then computed and the reliability calculated. If the Cronbach's alpha (α) value was acceptable (above 0.6 to 0.7) and could not be improved by the removal of items, this was acknowledged as a domain [28]. The process was carried out for all feasible domains (typically two or three per condition). The resulting domains were then correlated with all the remaining condition-specific items. An item was added to a domain if it correlated with a domain, it loaded only on one domain, and it generally made sense to include the item in the domain [29]. The reliability of the domain, including the added items, was then re-calculated to ensure a good fit. In some instances items were removed on the basis of low corrected item-total correlations, which ideally should be above 0.4 [28].

If the constructed domains displayed an unsatisfactory (depending on group size and number of items) Cronbach's alpha value (i.e. α below a value of 0.7 to 0.6), the factor analysis was repeated, restricting it to two or three domains. This typically resulted in the grouping of similar items that could be formed into possible new domains (not necessarily those identified in the card sorting procedure). If a domain contained too many items and had a very high alpha value (α over 0.9), item-item correlations were carried out to identify and consequently exclude duplicate items.

When two or three domains had been identified with a total of around 15 items, a final check was run that consisted of the reliability of the domain, the item-domain correlation, and conceptual analysis that included whether or not the scale made sense. The internal consistency of the domains in each condition-specific module was between 0.75 and 0.89 (Table 2). Each domain was given a label that represented the semantic content. Consultants (with knowledge of a specific chronic condition) within the DISABKIDS project were given the opportunity of adding 1 or 2 items to a module on the basis of clinical importance; these items were not added to the domains, but were maintained as single items for separate analyses after the field study.

**Table 2:** Domains, number of items (n) and the Cronbach's alpha (α) after the pilot analysis

**Condition**	**Domain 1**	**n**	**α**	**Domain 2**	**n**	**α**	**Domain 3**	**n**	**α**
Asthma	Impact	8	.83	Worry	5	.86	.	.	.
Juvenile idiopathic arthritis	Limitation	6	.82	Understanding	6	.75	Frustration	5	.77
Atopic dermatitis	Impact	7	.84	Skin	5	.77	Shame	4	.77
Cerebral palsy	Limitation	5	.84	Frustration	7	.81	.	.	.
Cystic fibrosis	Impact	6	.77	Treatment	8	.87	.	.	.
Diabetes mellitus	Impact	5	.84	Food	5	.76	Injections	5	.82
Epilepsy	Fear	8	.89	Social	6	.77	.	.	.

### Example: the asthma pilot study analysis

After the card sorting methodology the asthma module originally consisted of 8 domains (Limitations, Symptoms, Worry, Allergy, Sleep, Medical, Interpersonal and Lack of energy) with a total of 32 items. Analysis of the module as described above (including information from the cognitive interviews and clinical judgements) resulted in a 2 domain structure (13 items). The domains were labelled 'Impact' and 'Worry' due to their semantic content. The mean score on the 'Impact' domain was 3.63 (SD 0.82) and 4.15 (SD 0.89) on the 'Worry' domain. The DISABKIDS asthma consultants added two extra items, not selected through statistical analysis but based on clinical relevance.

### Field study

The next step in the DISABKIDS project was the field study (Figure 2), which took place between April and July 2003. The sample for the field study consisted of 1152 participating families. The field study instrument included the chronic generic module (56 items) [22] and the seven condition-specific modules (between 14 and 19 items) (Table 3). An equal number of boys and girls (52% vs. 48%) were included, mean age 12.2 (SD 2.8). The asthma group was the largest in the sample (n = 405). Data from 1094 children and adolescents were used in the analysis, selected on the basis of more than 60% of the items in the module being completed.

**Table 3:** Number of items and participants (n = 1152) in the field study for each condition-specific module

**Condition**	**Number of items**	**Number of participants**	**Percentage of total sample (%)**
Asthma	15	405	35
Juvenile idiopathic arthritis	19	150	13
Atopic dermatitis	19	65	5
Cerebral palsy	16	43	4
Cystic fibrosis	14	91	8
Diabetes mellitus	15	207	18
Epilepsy	16	191	17

At this stage the purpose of the analysis was to replicate the domains found in the pilot test analysis. Principal components analysis was carried out. Components that were found to be similar to the pilot test domains (like the asthma and CF module) were directly checked for reliability. A domain was kept if the alpha value was above 0.7 and could not be improved by the removal or inclusion of items.

All domains were correlated with each of the condition-specific items. An item was added to a domain if it correlated with the domain, it loaded clearly on one domain and it generally made sense to include the item in the domain. Items were removed if they loaded on more than one domain (above 0.4 for each domain) or on the basis of high item-item correlations (above 0.9) [29]. If necessary, items were also removed from a domain on the basis of low corrected item-total correlations and/or a substantial increase in alpha value if removed. The internal consistency of the domains was checked after each step. Each procedure was repeated until the optimal solution was found. In some cases domains were renamed or two domains were merged (for example for the diabetes, JIA, and atopic dermatitis modules). The internal consistency of the domains for each condition-specific module was between 0.71 and 0.90 (Table 4). It became clear that one domain of each condition related to the actual impact of the condition on a child or adolescent's life. These domains were relabelled 'Impact'. Over half of the extra items that were included on the basis of clinical relevance after the pilot study analysis were integrated in the final domains.

**Table 4:** Domains, number of items (n) and Cronbach's alpha (α) after the field study analysis

**Condition**	**Domain 1**	**n**	**α**	**Domain 2**	**n**	**α**
Asthma	Impact	6	.83	Worry	5	.84
Juvenile idiopathic arthritis	Impact	9	.87	Understanding	3	.73
Atopic dermatitis	Impact	8	.87	Stigma	4	.71
Cerebral palsy	Impact	10	.82	Communication	2*	.72
Cystic fibrosis	Impact	4	.80	Treatment	6	.85
Diabetes mellitus	Impact	6	.83	Treatment	4	.84
Epilepsy	Impact	5	.90	Social	5	.84

*With only two items this is the inter-item correlation.

### Example: the asthma field study analysis

The domain structure of the asthma pilot test analysis was successfully replicated resulting in a 2 domain structure of 'Impact' and 'Worry', which consist of 6 and 5 items respectively. Four items were removed on the basis of duplication and low item-domain correlations, including the two extra clinical items. The cumulative proportion of the variance explained by the first two domains was 53% and the internal consistency (α) was 0.83 and 0.84 (Table 4). The mean score on the 'Impact' domain was 3.61 (SD 0.91) and 4.17 (SD 0.84) on the 'Worry' domain. The asthma-specific module was tested separately for all participating DISABKIDS countries. The reliability in each country was mostly above 0.8 (Table 5).

**Table 5:** The Cronbach's alpha (α) and number of participants (p) for the final two asthma-specific domains calculated for each country

**Asthma**	**Impact α**	**p**	**Worry α**	**p**
Austria	.77	30	.80	30
France	.84	36	.77	34
Germany	.91	38	.88	41
Greece	.72	29	.61	38
Netherlands	.81	122	.84	127
Scotland	.86	48	.84	49
Sweden	.85	72	.86	73

## Discussion

This study describes part of the development process of the seven DISABKIDS cross-national condition-specific modules. The DISABKIDS instrument for children and adolescents is the first to be developed cross-nationally in collaboration with several European countries and to include a chronic generic and condition-specific module.

The DISABKIDS instrument has several advantages. First the construction of the chronic generic and condition-specific modules allows for a comprehensive assessment of HRQoL. The chronic generic module can be used in conjunction with any of the condition-specific modules. Combining these modules gives the clinician and investigator the unique opportunity to compare between countries and between different conditions.

The second advantage is the simultaneous cross-national patient-derived development of the DISABKIDS instrument. Children and adolescents from each DISABKIDS country were included in the developmental process of the instrument. HRQoL statements were collected from the cross-national focus groups and interviews. Investigators from the DISABKIDS centres were involved in the item selection process, assuring that all items where relevant in each country. This was again tested in the cognitive interview in the pilot study. This simultaneous setup in different countries supported the developmental process by taking into account cross-national consensus on important HRQoL issues.

In addition, the construction of the DISABKIDS instrument has been a reflective one, combining subjective and statistical procedures. Item selection and reduction was not carried out solely through the use of statistical methods, but also through the inclusion of qualitative factors, such as the views of children and adolescents (gained from cognitive interview) and clinical judgement. The domain structure that resulted from the pilot test was to a great extent successfully replicated after the field test. The reliability of each domain was satisfactory in each condition-specific module.

However, some limitations should be given consideration. The number of respondents in some condition groups in both the pilot and the field test was relatively small, CP (n = 21 and 43) and atopic dermatitis groups (n = 29 and 65) in particular (Table 1 and 3). It was therefore not possible to solely use statistical methods to develop these modules. It is important to carry out further data collection and to test the reliability and validity in larger patient groups for these conditions. It will also be necessary to carry out large cross-national studies in the future in order to use modern psychometric methods based on Item Response Theory (IRT), which will permit the testing of differential item functioning across cultures and inform the degree to which cross-national comparisons can be validly made. The use of such IRT-based tests was not possible at this stage of the development of the measure because IRT methods require very large sample sizes.

A second limitation is that the condition-specific modules were not tested in every country. Only asthma was tested in all the participating DISABKIDS countries. The Cronbach's alphas were adequate for each asthma domain in each country. The lower alphas in Greece might not only be due to lower numbers of tested participants but also to the fact that the researched population included mostly exercise-induced asthma, which might result in a different impact on their HRQoL. As the number of participants in the other chronic conditions was generally low the reliability per country will still need to be explored in more detail.

Future studies will be necessary to provide more details on the reliability and validity of the DISABKIDS modules, especially in larger groups and in different countries. Evidence also needs to be supplied on the value of the instrument in clinical practice. Further possibilities include testing the chronic generic module for applicability in other chronic medical conditions (e.g. haemophilia, heart disease or obesity).

The developmental steps within the DISABKIDS project have included a combination of qualitative and quantitative methods. The two methods were used in succession in order to complement each other, as has been the case throughout the DISABKIDS project. The qualitative data (cognitive interview and clinical judgement) collected in the pilot study was first used to disregard irrelevant items. This was followed by the psychometric calculations. In some cases the project members found removed items to be clinically relevant. These were therefore added as the two extra items in the field study.

Although the process of item reduction for each of the condition-specific modules was similar and included well know procedures [28,29], it remains difficult to describe the developmental process. As the value of each test depended on the size of the group and the number of items in the domain, and common sense judgements were also included, the taken steps may not always seem transparent. The number of countries included in the study meant that there were more national factors and individual opinions to include. Several processes within the DISABKIDS project (team meetings, group discussions) have influenced decisions. An example was the post-hoc decision to add extra items based on clinical relevance.

## Conclusion

The condition-specific modules for the DISABKIDS instrument were developed through a step-by-step process including cognitive interview, clinical expertise, factor analysis, correlations and reliabilities. The seven condition-specific modules consist of an 'Impact' domain and an additional domain with a total of 10 to 12 items.

The DISABKIDS project has constructed a unique instrument, which was developed cross-nationally, included the patient's perspective and has a chronic generic module, which can be combined with one of the seven condition-specific modules. The expectation is that the instrument will be used in a wide variety of (international) studies of children and adolescents with common disorders of childhood.

## Authors' contributions

R.M. Baars was one of the asthma consultants in the DISABKIDS project, participated in the data collection and was responsible for the selection of items and analysis of the condition-specific modules. She performed the literature research, data analyses and writing of the manuscript. C. I. Atherton was the cerebral palsy consultant in the DISABKIDS project, participated in the data collection and was responsible for the analysis of the condition-specific modules. She performed the literature research, data analyses and writing of the manuscript.

R.M. Baars and C. I. Atherton both participated equally in the development of the condition-specific module and the writing of the manuscript. H.M. Koopman was also one of the asthma consultants in the DISABKIDS project. He participated in all the research steps and worked on the manuscript. M.Bullinger coordinated the DISABKIDS project. She contributed to all stages of the instrument development and as revised the manuscript. M. Power was a principal investigator in the DISABKIDS project. He participated in all the research phases and advised RMB and CA during the statistical analysis of the condition-specific modules and revised the manuscript. All authors read and approved the final manuscript.

All members of the DISABKIDS group were included in each step taken in the European project and contributed in meetings and by testing the DISABKIDS instrument in their country. All members have received the manuscript and have had the opportunity to give feedback or implement changes.

## Supplementary Material

Additional file 1 1. Illustration of the item selection and domain appointment in the asthma module. 2. The items and domains of the DISABKIDS condition-specific modules. 3. Summary of the analysis steps Click here for file
