# Supplementary material for: The European DISABKIDS project: development of seven condition-specific modules to measure health related quality of life in children and adolescents
Source: Health Qual Life Outcomes. 2005 Nov 13;3:70. doi: 10.1186/1477-7525-3-70 (PMC1326227; doi:10.1186/1477-7525-3-70)
Supplement: Additional file 1 — 1. Illustration of the item selection and domain appointment in the asthma module. 2. The items and domains of the DISABKIDS condition-specific modules. 3. Summary of the analysis steps [file 1477-7525-3-70-S1.doc]

**Additional files**

1 - Illustration of the item selection and domain appointment in the asthma module

| **Asthma condition-specific items** | **Pilot study domains** | **Field study domains** | **Final domains** |
| --- | --- | --- | --- |
| Do you have problems sleeping at night because of your cough? | Sleep | *Extra item* |  |
| Are you bothered by coughing during your sleep? | Sleep |  |  |
| Are you scared at night because of your asthma? | Sleep | Worry | Worry |
| Does coughing give you attention from people? | Interpersonal |  |  |
| Do other children understand that you are sometimes out of breath? | Interpersonal |  |  |
| Do other kids make fun of your inhaler? | Medication |  |  |
| Do you hate blowing into a peak flow meter? | Medication |  |  |
| Do you need medicine to relieve your symptoms before going to bed? | Medication |  |  |
| Do you worry that others do not know what to do if you have an asthma attack? | Worry | Worry | Worry |
| Are you worried that you might have an asthma attack? | Worry | Worry | Worry |
| Do you feel scared that you might have difficulty breathing? | Worry | Worry | Worry |
| Are you scared that you might have to go to the emergency ward? | Worry | Worry | Worry |
| Are you bothered that you have to stay indoors because of your allergies? | Worry |  |  |
| Do you have to ask people not to smoke or to wear perfume? | Allergy |  |  |
| Do you have to wear special clothes because of your asthma? | Allergy |  |  |
| Are you bothered by hay fever? | Allergy |  |  |
| Do you have to be careful about washing yourself due to the eczema? | Allergy |  |  |
| Are you bothered by feeling sleepy? | Lack of energy |  |  |
| Do you feel that you get easily exhausted? | Lack of energy | Impact | Impact |
| Do your parents prevent you from going out as much as your friends because of your asthma? | Limitations |  |  |
| Does asthma bother you if you want to go out? | Limitations | Impact | Impact |
| Do you avoid going to people’s houses in case they are not clean enough? | Limitations |  |  |
| Are you not able to take part in certain sports? | Limitations | Impact | Impact |
| Do you miss having a pet? | Limitations |  |  |
| Do you miss cuddly toys? | Limitations |  |  |
| Does your allergy stop you from doing what you want to do? | Limitations | *Extra item* |  |
| Do you feel terrible when you are out of breath? | Symptoms | Impact | Impact |
| Do you feel short of breath when you do sports? | Symptoms | Impact | Impact |
| Are you bothered by the amount of time you spend wheezing? | Symptoms | Impact | Impact |
| Are you bothered by the amount of time you spend coughing? | Symptoms | Impact |  |
| Have you been embarrassed about coughing in front of others? | Symptoms |  |  |
| Do you cough when you do sports? | Symptoms | Impact |  |

2 - Summary of the analysis steps

| **Exploring the data**  Select data for each condition separately  Remove children with more than 40% missing on condition-specific module  Descriptives including mean, SD, missings, skewness, and kurtosis  Check total domain alpha and each item’s corrected item-total correlation  Check item-item correlations for high or not correlating items  **Checking existing domains**  Compute original scales from card sorting  Reliability of existing scales  **Replicating domain structure**  Factor analysis, if necessary restricted to four, three or two factors  Comparing meaningfulness of solutions  Computing domains, calculating reliability and the item-domain correlation  Adding any correlated items to domains  Item-domain correlations  **Testing domains.**  Total reliability of the domains  Checking item-domain correlations (ensure corrected item-total correlation is above 0.4)  Domain-domain correlation  Check deleted items against content of chronic generic items |
| --- |

3 - The items and domains of the DISABKIDS condition-specific modules.

| Asthma | |
| --- | --- |
| Impact  Worry | Do you feel that you get easily exhausted?  Does asthma bother you if you want to go out?  Are you unable to take part in certain sports?  Do you feel short of breath when you do sports?  Are you bothered by the amount of time you spend wheezing?  Do you feel terrible when you are out of breath?  Are you worried that you might have an asthma attack?  Do you worry that others do not know what to do if you have an attack?  Do you feel scared that you might have difficulty breathing?  Are you scared that you might have to go to the emergency ward?  Are you scared at night because of your asthma? |
| Juvenile idiopathic arthritis | |
| Impact  Understanding | Do you feel stiff in the mornings (like an old grandma/granddad)?  Do you get exhausted easily?  Does arthritis make you feel too exhausted to be with friends?  Do you hate being in pain?  Does it annoy you that the pain sometimes comes on so suddenly?  Does pain stop you from doing what you want?  Does it bother you that you can’t do all sports/hobbies because of your arthritis?  Do you hate being restricted in movement?  Does it bother you that you have trouble writing/ drawing?  Do others understand that your symptoms may change suddenly?  Do your friends understand that you may feel poorly quite suddenly?  Do teachers understand that you sometimes can’t join in? |
| Atopic dermatitis | |
| Impact  Stigma | Does the itching bother you?  Does the appearance of your skin bother you?  Does itching bother you during the night?  Does your skin condition affect your concentration at school?  Does looking at your skin scare you?  Does your skin get worse when you are under stress?  Does your skin condition affect your free-time (sports, playing)?  Do you feel comfortable with the way your skin is?  Do you try to hide your skin condition?  Are you annoyed by others giving you strange looks?  Do you dislike it when your friends see the cream being applied?  Do you feel uncomfortable when others look at you? |
| Cerebral palsy | |
| Impact  Communication | Is it frustrating to be unable to keep up with other children?  Do you wish that you could run around like everyone else?  Do you wish that you could swim as well as other children?  Does it bother you that getting dressed takes a long time?  Do people think that you are not as clever as you are?  Do you have trouble getting in and out of buildings?  Are you able to do most things even though your legs don’t move well?  Does it upset you that you are unable to walk without help?  Do you dislike being washed and dressed by other people?  Does it upset you that you need help to use the toilet?  Can you communicate as well as you’d like?  Does it upset you that you can’t talk as well as other children? |
| Cystic fibrosis | |
| Impact  Treatment | Do you get exhausted when you do sports?  Do you feel tired during the day?  Do you get out of breath?  Do you need to rest more than others?  Does it bother you that you must take your enzymes before every meal?  Does it bother you that you have to eat a special diet to keep you healthy?  Does it bother you that you have to spend a lot of time having treatment?  Are you bothered because you have to do physiotherapy everyday?  Have you felt that your treatment takes up too much of your free time?  Do you feel bothered that you have to stop playing or doing things for treatment? |
| Diabetes mellitus | |
| Impact  Treatment | Does diabetes stop you from doing the things you want to do?  Does diabetes rule your day?  Does it bother you that you have to be careful about what you eat?  Is it difficult for you to stick to your diet?  Do you worry about your blood sugar level?  Does it bother you that others can always eat and drink as much as they like?  Are you annoyed that you have to carry the testing equipment with you?  Are you bothered that you have to plan everything?  Do you mind taking insulin?  Do you get fed up with measuring your blood sugar levels? |
| Epilepsy | |
| Impact  Social | Are you afraid that you might hurt yourself during a seizure?  Are you worried that you might have a seizure in public?  Are you afraid of having a seizure?  Do your seizures make you feel helpless?  Are you scared that you could have a seizure at any time?  Does it embarrass you when people take care of you when you have a seizure?  Are you worried that people make fun of you when you have a seizure?  Are you afraid that you can’t remember what happens during a seizure?  Are you ashamed of having seizures?  Are you worried that other children will see you having a seizure? |
